# Supplementary material for: Preparing for Cardiopulmonary Bypass: A Simulation Scenario for Anesthesia Providers
Source: MedEdPORTAL. 2017 May 8;13:10578. doi: 10.15766/mep_2374-8265.10578 (PMC6338152; doi:10.15766/mep_2374-8265.10578)
Supplement: Supplementary file 1 — A. Simulation Case.docx B. Supplemental Data.docx C. Critical Actions Checklist.docx D. Debriefing Summary.docx E. Evaluation Form.docx [file mep-13-10578-s001.zip › E. Evaluation Form.docx]

**Appendix E- Evaluation Form _ Cardiopulmonary Bypass**

Date: Faculty Facilitator:

Level of training of learner:

Please rate the simulation using the following statements: (1 is strongly disagree, 5 is strongly agree)

**The facilitator helped me to recognize my areas of strength discovered during this simulation**

Strongly Disagree Somewhat Disagree Neutral Somewhat Agree Strongly Agree

1 2 3 4 5

**The facilitator helped me to identify areas for improvement discovered during this simulation**

Strongly Disagree Somewhat Disagree Neutral Somewhat Agree Strongly Agree

1 2 3 4 5

**The facilitator presented the content clearly in a manner easy to understand**

Strongly Disagree Somewhat Disagree Neutral Somewhat Agree Strongly Agree

1 2 3 4 5

**The content was current and relevant to my practice**

Strongly Disagree Somewhat Disagree Neutral Somewhat Agree Strongly Agree

1 2 3 4 5

**This activity was appropriate for my level of education and training**

Strongly Disagree Somewhat Disagree Neutral Somewhat Agree Strongly Agree

1 2 3 4 5

**This simulation enhanced my understanding of how to manage critically ill patients in the perioperative period**

Strongly Disagree Somewhat Disagree Neutral Somewhat Agree Strongly Agree

1 2 3 4 5

**Comments:**
