# Supplementary material for: Preparing for Cardiopulmonary Bypass: A Simulation Scenario for Anesthesia Providers
Source: MedEdPORTAL. 2017 May 8;13:10578. doi: 10.15766/mep_2374-8265.10578 (PMC6338152; doi:10.15766/mep_2374-8265.10578)
Supplement: Supplementary file 1 — A. Simulation Case.docx B. Supplemental Data.docx C. Critical Actions Checklist.docx D. Debriefing Summary.docx E. Evaluation Form.docx [file mep-13-10578-s001.zip › _Educational Summary Report_10578.pdf]

MedEdPORTAL®
The Journal of Teaching and Learning Resources

Original Publication

OPEN ACCESS

Preparing for Cardiopulmonary Bypass: A Simulation Scenario for Anesthesia Providers

Brett Myers, MD*, Clark Obr, MD
*Corresponding author: brett-a-myers@uiowa.edu

Citation: Myers B, Obr C. Preparing for cardiopulmonary bypass: a simulation scenario for anesthesia providers. *MedEdPORTAL*. 2017;13:10578. https://doi.org/10.15766/mep_2374-8265.10578

Copyright: © 2017 Myers and Obr. This is an open-access article distributed under the terms of the Creative Commons Attribution license.

Appendices

A. Simulation Case.docx
B. Supplemental Data.docx
C. Critical Actions Checklist .docx
D. Debriefing Summary.docx
E. Evaluation Form.docx

All appendices are peer reviewed as integral parts of the Original Publication.

Abstract

Introduction: This simulation on cardiopulmonary bypass (CPB) introduces learners to the relevant critical actions and concepts involved in going onto and off of CPB. It is intended that junior residents experience this simulation immediately prior to beginning their cardiac anesthesia rotation. Thus, this simulation serves to segue to the trainee's initial clinical experience with CPB. **Methods:** The case is fully presented for facilitators in the simulation case file, which includes a brief narrative description of the case, learning objectives to be covered by the simulation, and a summary of critical actions to be performed by the learner during the educational activity. It is optimal to run this simulation using a high-fidelity human patient simulator with anesthesia machine and relevant monitors. **Results:** The simulation was carried out by eight CA-1 or CA-2 residents during the 2016 academic year. Cardiac anesthesia faculties observed a significantly improved learning curve for trainees who had experienced this simulation prior to their first clinical case. **Discussion:** Overall, this simulation has been found to be a very effective learning tool at the University of Iowa. To that end, this simulation has been incorporated into the cardiac anesthesia curriculum, and all junior residents experience this simulation prior to beginning their rotation.

Keywords

Cardiac Anesthesia, Cardiopulmonary Bypass, Anticoagulation for Cardiac Bypass

Educational Objectives

By the end of this session, learners will be able to:

1. Outline the critical actions to successfully go on cardiopulmonary bypass (CPB).
2. Outline the critical actions to successfully come off of CPB.
3. Outline the goals of anticoagulation prior to initiating CPB.
4. Discuss protamine and its potential adverse effects.

Introduction

Cardiopulmonary bypass (CPB) is a common procedure performed in the operating room to assist cardiothoracic surgeons while they work by maintaining systemic circulation of blood. It is a very unique process with potential untoward consequences. Thus, a thorough understanding of the principles involved in initiation and discontinuation of CPB is necessary.

For the junior trainee with no experience in cardiac anesthesia, it is often a daunting task to assimilate the concepts of managing a CPB case. With that in mind, we created this simulation to introduce these concepts to the resident prior to his or her first cardiac anesthesia clinical case. This simulation is based on the generalized goals identified in the literature as well as on institutional experience at the University of Iowa. The simulation was instituted at the University of Iowa for resident physicians within the Department of Anesthesiology. Learners from the residency program are required to complete several months of clinical anesthesia prior to beginning their cardiac anesthesia rotation.

This simulation is designed to be a single 1-hour session using the adult SimMan (Laerdal Medical) medical simulator connected to standard monitors. The simulation can be proctored by a single instructor with the help of a technician for the high-fidelity medical simulator. Vital signs are provided throughout the

simulation via the monitors connected to the simulator; laboratory results are provided to the learner by the instructor. In addition, a pediatric manikin suitable for practicing resuscitation is necessary for the final phase of the simulation. By using the SimMan medical simulator to provide real-time vital signs and procedural guidance, learners are immersed in this experiential learning opportunity rather than simply participating in a didactic learning session, which in turn improves the overall learning experience.[1,2]

Methods

The case is fully presented for facilitators in the simulation case file (Appendix A), which includes a brief narrative description of the case, the learning objectives to be covered by the simulation, and a summary of critical actions to be performed by the learner during the educational activity. In addition, this file also contains information on the simulated patient's initial presentation, including history of previous illness, vital signs, physical exam findings, and laboratory studies, and a guide as to how the case is to progress based on the actions taken by the learner. The file concludes with a sample of ideal management of the simulated patient as well as some common pitfalls that have been demonstrated by trainees while performing the simulation.

Optional supplemental materials (Appendix B) are available at the request of the learner during the simulation. Each item is noted as to whether it is provided upon request, spontaneously, or when the learner performs a certain action.

A separate critical actions checklist (Appendix C) is included for learners to reference while running the simulation. The checklist also contains a tool by which the instructor can rate the learner based on performance on the primary learning objectives.

The debriefing summary (Appendix D) contains a written summary of the pathophysiology and treatment guidelines used to govern the simulated case. It is designed to be read both by the instructor prior to the case in order to refresh critical concepts and by the learner after the case to solidify learning points discovered during the simulation. To this end, the summary can be printed as a handout to be given to the trainee following the simulation.

The evaluation form (Appendix E) allows the learner to provide feedback regarding the simulation. The evaluation form addresses feedback both for the facilitator who leads the simulation and for the content of the simulation itself.

For most learners, experiencing a high-acuity situation such as CPB in real time in the low-risk environment of a high-fidelity simulation center is a much more effective learning experience than simply reading about the process and eventually being thrust into a clinical case. The experiential learning afforded by this simulation better prepares trainees for their initial clinical case involving CPB.

Equipment/Environment

Because this simulation is designed to be performed on a high-fidelity simulator for improved efficiency and realism, access to a SimMan or other similar medical simulator is beneficial. Other optimal equipment includes simulated real-time vital sign monitoring with cuff and arterial blood pressures, capnograph, pulse oximeter, EKG, and temperature probe; airway supplies including laryngoscope, supraglottic airways, and endotracheal tubes for securing adult and pediatric manikin airways; a variety of preprepared syringes including propofol, etomidate, succinylcholine, rocuronium, fentanyl, lidocaine, ketamine, midazolam, ephedrine, and phenylephrine to simulate medication administration; and a CPB circuit if available. The environment is set up to simulate that of an operating theatre with all associated supplies, again to lend a degree of realism to the simulation.

Personnel

In addition to the instructor facilitating the simulation, it is beneficial to have a technician for the high-fidelity simulator to provide changes in patient status and vital signs in real time. It is recommended that the facilitator and technician discuss the case in its entirety before the simulation is performed to improve

communication and coordination between team members. A perfusionist's presence would be beneficial but is not required. The aim of this simulation is to understand not the CPB circuit but rather the critical actions and thought processes required in going on and coming off CPB.

Assessment
Learners are to be assessed on items that are critical to quality of care of the patient undergoing CPB. Thus, we created a critical actions checklist. The goal of the simulation is for the trainee to develop an understanding of these critical actions and the logical progression through each step. The junior trainee will require guidance through these critical actions, and the simulation serves as a learning tool to provide him or her with real-time training on management of a CPB case. For the advanced resident, the simulation serves as a refresher regarding the critical actions necessary in a CPB case.

Debriefing
Following the completion of this simulation, it is recommended that the learner first be given the opportunity for self-reflection with questions such as "Overall, how did you think the case went?", "What do you think you did well?", and "What do you think is an area where you could improve?" The instructor then discusses the critical actions checklist with the learner, stopping periodically to ask questions about pathophysiology that encourage discussion and further learning. When the discussion is complete, the pathophysiology and perioperative management of CPB are written up in the debriefing summary, which can also be presented to the learner as a handout.

Results

During the 2016 academic year, eight anesthesiology resident physicians experienced the content of this simulation. The targeted learners at our institution were CA-1 or CA-2 residents immediately prior to their first cardiac rotation. The simulation was presented by or with the assistance of the core simulation faculty within the Department of Anesthesiology. We have found that experienced CA-3 residents or fellows are effective in facilitating this simulation with junior trainees. Overall, the learners found this to be a valuable learning experience prior to entering their specialty cardiac anesthesia training. Responses from the learners were that this was a very helpful introduction to CPB and that it should be implemented in the curriculum. The responses on the feedback forms provided to the learners were all in the range of *agree* or *strongly agree.*

After experiencing this simulation, all learners stated they felt more comfortable entering their first cardiac case. Faculty evaluation of the trainees indicated that the primary educational objectives of this simulation were achieved: Junior trainees in their initial clinical cases were more comfortable and exhibited a smaller learning curve with regard to knowledge of the critical actions associated with going onto and off of CPB, as well as goals of anticoagulation for CPB and use of protamine. The scenario and checkpoints were created specifically for our institution's common protocols. We do not have resident evaluation data for trainees prior to the incorporation of this simulation into the curriculum. However, cardiac anesthesia faculty consistently noted that trainees who had experienced this simulation were, in general, more confident and had an improved learning curve as they began their cardiac anesthesia rotation. Multiple staff members commented that the junior residents were more prepared on their first day of cardiac anesthesia training.

Discussion

This simulation was created to provide learners with the opportunity to manage a CPB case in a high-fidelity, low-risk environment. The opportunity to create this learning experience arose out of a recognized gap in simulation training on this particular issue. Introducing trainees to the basic concepts and critical actions of a CPB case immediately prior to the start of their cardiac anesthesia rotation allows for a smaller learning curve during their initial clinical cardiac anesthesia cases. This simulation is administered in conjunction with another 1-hour class on the CPB circuit given by the perfusionists at our institution.

There has been little adjustment to the scenario as most of the deficiencies of the learner were recognized prior to implementation. We did decide to introduce the learner to a pulmonary artery catheter

for further learning associated with the simulation. As would be expected, we also found that the simulation was less beneficial when the trainee had not done the required preliminary reading. When the learners were too early in their training, it was found to be beneficial to first guide them through the stages of initiating and separating from CPB, approaching very broad goals, and then going back through the actual simulation.

The educational material provided to the junior learners prior to the simulation was found to be very helpful in creating a dialogue when asking them specific questions about anticoagulation and the effects of protamine. This was obviously a very truncated discussion of the pharmacology of these medications, but it seemed to be at an appropriate depth for introducing the intended audience to the topic at this stage in their training. The overall goal of introducing learners to these drugs and the importance and dangers of each in the cardiac arena was fulfilled by these discussions. Learners could then readdress the topic in greater breadth at a later date.

Overall, this simulation has been found to be a very effective learning tool at the University of Iowa. To that end, our faculty incorporated this simulation into the cardiac anesthesia curriculum, and all junior residents experience this simulation prior to beginning their rotation. The simulation can be expanded and tailored to more senior trainees as well. For example, adding more complicated patient pathology with hemodynamic and coagulation derangements or focusing on the nuances of specific aspects of CPB would benefit the knowledge base of senior trainees.

Initiating and separating from CPB in a simulation setting are difficult at our institution as we do not have the physical setup or equipment necessary to fully exemplify this scenario. This places a fair amount of responsibility on the facilitator to explain some of the intricacies of the scenario as well as to point out any learning objectives that are not obvious due to our lack of facilities or equipment. To further help with this, we have a separate 1-hour simulation facilitated by a cardiac anesthesiologist with the perfusion team and a junior learner to further acclimate trainees to the cardiac arena.

Brett Myers, MD: Chief Resident, Department of Anesthesiology, University of Iowa Roy J. and Lucille A. Carver College of Medicine

Clark Obr, MD: Clinical Associate Professor, Department of Anesthesiology, University of Iowa Roy J. and Lucille A. Carver College of Medicine

Disclosures
None to report.

Funding/Support
None to report.

Ethical Approval
Reported as not applicable.

References

1. Krage R, Erwteman M. State-of-the-art usage of simulation in anesthesia: skills and teamwork.*Curr Opin Anaesthesiol.* 2015;28(6):727-734. https://doi.org/10.1097/ACO.0000000000000257
2. Wallace A. Cardiac anesthesiology made ridiculously simple. Cardiac Engineering Web site. http://www.cardiacengineering.com/cardiaca.htm. Retrieved March 1, 2016.

Received: December 19, 2016 | **Accepted:** April 14, 2017 | **Published:** May 8, 2017
