# Supplementary material for: Preparing for Cardiopulmonary Bypass: A Simulation Scenario for Anesthesia Providers
Source: MedEdPORTAL. 2017 May 8;13:10578. doi: 10.15766/mep_2374-8265.10578 (PMC6338152; doi:10.15766/mep_2374-8265.10578)
Supplement: Supplementary file 1 — A. Simulation Case.docx B. Supplemental Data.docx C. Critical Actions Checklist.docx D. Debriefing Summary.docx E. Evaluation Form.docx [file mep-13-10578-s001.zip › A. Simulation Case.docx]

| **Appendix A: Simulation Case Cardiopulmonary Bypass**  **SIMULATION CASE TITLE:** Cardiopulmonary Bypass: A Cardiac Simulation Case for Anesthesiology Residents  **AUTHORS:** Brett Myers MD, Clark Obr MD. | |
| --- | --- |
| **PATIENT NAME: Mike Baker**  **PATIENT AGE: 47 year old male**  **CHIEF COMPLAINT: Bicuspid Aortic Valve** | |
|  | |
| **Brief narrative description of case** | You have successfully anesthetized a 47 yo male with a history of bicuspid aortic valve with critical aortic stenosis on echo with no other PMH. Currently on a 0.05 mcg/kg/min norepinephrine infusion with stable hemodynamics and appropriate amount of anesthetic for an aortic valve replacement. You are about to initiate cardiopulmonary bypass. The goals for the case are to successfully navigate initiation and separation from cardiopulmonary bypass while identifying critical actions necessary to achieve safely achieve this. |
| **Primary Learning Objectives** | 1. Introduce junior residents to the basic steps of going on and coming off cardiopulmonary bypass.   (Anesthesiology Milestones: Medical Knowledge 1; Practiced-based learning and Improvement 2, 3)   1. Outline the steps to successfully go on cardiopulmonary bypass.   (Anesthesiology Milestones: Medical Knowledge 1; Practiced-based learning and Improvement 2, 3)   1. Outline the steps to successfully come off cardiopulmonary bypass.   (Anesthesiology Milestones: Medical Knowledge 1; Practiced-based learning and Improvement 2, 3)   1. Outline the goals of anticoagulation for initiating and maintaining cardiopulmonary bypass.   (Anesthesiology Milestones: Medical Knowledge 1; Practiced-based learning and Improvement 2, 3)   1. Discuss protamine and its potential adverse effects.   (Anesthesiology Milestones: Medical Knowledge 1; Practiced-based learning and Improvement 2, 3) |
| **Critical Actions** | - - - Prior to Bypass       - Baseline Activated Clotting Time (ACT)       - Heparinization – know to withdraw blood and give heparin centrally       - Redraw ACT at appropriate interval after heparin       - Confirm ACT appropriate for CPB       - Systolic Blood Pressure (SBP) <100 prior to aortic cannulation (optional)       - Empty urometer for bypass       - Watch circuit for clot       - Machine Alarms to bypass mode       - Turn Volatile gas off/ventilator off. Ensure perfusion has appropriate volatile on board       - Turn vasoactive infusions off when Mean Arterial Pressure (MAP) stabilizes on bypass       - Other IV anesthetics to administer?       - Optional – verify cannulation with (Transesophageal Echocardiogram) TEE       - Verify appropriate blood product availability     - On bypass       - Watch MAP, urine output, ACT       - Optional – observe Bispectral index and cerebral oximetry       - Treat hyperglycemia       - Do I need further coagulation testing or thromboelastogram prior to separation from bypass       - Reassess availability/necessity for blood product       - Patient’s heart rhythm? Pacing required? Do you have a pacing box? Did you check the batteries?     - Coming off Bypass       - What is patient’s temperature? Observe the difference between peripheral and core temperature       - Check arterial blood gases, electrolytes, acid base status, hemoglobin as needed       - Patient’s heart rhythm? Pacing required?       - Are you ventilating?       - Is there air in the heart on echo? Do we need a Valsalva/Trendelenburg to de-air the heart       - Is the monitor switched off Cardiac Bypass Mode?       - Zero your invasive pressure monitors       - Do you have anesthetic on?       - Inotropy? SVR? How does the heart function look on TEE? Restart vasoactive infusions as necessary     - After Bypass       - Separated from cardiopulmonary bypass and hemodynamics are stable, protamine?       - Loudly announce to surgeon initiation of protamine       - Loudly announce 50% of protamine is in. Ask surgeon permission to proceed       - ACT normalized, send coagulation factors |
| **Learner Preparation** | Trainees will likely be exposed to at least 5 months of anesthesia at this time, but prior to cardiac anesthesia. The Trainee should have been provided with reading materials about cardiac anesthesia basics. http://www.cardiacengineering.com/cardiaca.htm |

| Initial Presentation | | | |
| --- | --- | --- | --- |
| **Initial vital signs** | HR 75; BP 110/55; RR 13; Temp 36.4^o^C; SpO2 100% on 50 fi02. | | |
| **Overall Appearance** | Anesthetized 47 yo 80 kg male. Hemodynamically stable, neuromuscular paralysis, 0.05 mcg/kg/min norepinephrine infusion, preparing for cardiopulmonary bypass. | | |
| **Actors and roles in the room at case start** | In addition to the learner, the instructor stands in the room to run the simulation as cardiac anesthesia staff and surgeon when needed, and the technician is out of sight controlling the high fidelity simulator. No additional actors or agents are necessary. Ideally a perfusionist would be present. | | |
| **HPI** | 47 yo male with new onset shortness of breath, syncope and intermittent angina found to have critical aortic stenosis and bicuspid aortic valve requiring replacement. He is currently anesthetized and hemodynamically stable. No other PMH or PSH. | | |
| **Past Medical/Surgical History** | **Medications** | **Allergies** | **Family History** |
| See above. | Metoprolol 25 mg daily | None | None |
| **Physical Examination** | | | |
| **General** | Alert, active, healthy appearing adult male | | |
| **HEENT** | Normal facies, normal airway exam | | |
| **Neck** | Normal neck flexion/extension, normal thyromental distance. | | |
| **Lungs** | Clear to auscultation bilaterally | | |
| **Cardiovascular** | Regular rate and rhythm, 3/6 systolic murmur radiating to the carotid arteries auscultated best at right second intercostal space | | |
| **Abdomen** | Abdomen soft, non-distended, non-tender. Normal bowel sounds. | | |
| **Neurological** | Alert, no focal deficits appreciated | | |
| **Skin** | Normal skin turgor, no rashes or lesions noted. | | |
| **GU** | deferred | | |
| **Psychiatric** | Appropriate for age | | |

| Instructor Notes - Changes and CASE Branch Points | | | |
| --- | --- | --- | --- |
| State | Patient Status | Student learning outcomes or actions desired and trigger to move to next state | |
| Phase I:  Preparation for CPB | Patient anesthetized, arterial line in right radial artery, Sheath introducer with pulmonary artery catheter in right internal jugular vein, 14g IV in right forearm:  HR 75, NSR  BP 110/54  RR 14  SpO2 100% on 50% fi02 | **Learner Actions:**  Learner may review patient history, including history of airway, cardiovascular, pulmonary, and neurologic exams.  Learner may inquire about line placement.  Learner may inquire about current anesthetic.  Learner may have asked for type and screen and blood to be in the room.  Learner should inquire about baseline ACT.  Learner should administer heparin when surgeon (facilitator) requests it.  Learner should recheck ACT (500 seconds) and verify adequacy of heparinization after appropriate duration of time.  Learner should optimize pressure for aortic cannulation.  Learner should check for emboli in circuit.  Learner should turn off ventilator, adjust monitors and turn off anesthetic.  Learner should verify perfusionist has anesthetic on.  Learner should turn off drips when MAP stabilizes on bypass. | **Operator:**  Begin scenario.  **Trigger:**  Learner proceeds with anesthetic and prepares for cardiopulmonary bypass. Surgeon (facilitator) will announce need for heparin as well as timing of aortic cannulation.  First dose of heparin will result in adequate ACT for CPB.  **Teaching Points:**  Appropriate steps for CPB.  Heparinization goals and potential complications.  Where to give heparin, quiz learner on where and how to administer (ie. Centrally and confirm intravascular line placement by withdrawing blood). |
| Phase II:  CPB | MAP 65  RR 14  SpO_2_ 100% | **Learner Actions:**  Learner should check urine output.  Learner should monitor electrolytes and blood glucose. | **Operator:**  Discuss CPB maintenance and expectations  **Trigger:**  Scenario, patient’s blood sugar 310 with no appreciable changes by anesthesia.  **Teaching Points:**  Discuss the effects of cardioplegia and its components. Discuss the effects of cardiopulmonary bypass on the coagulation system. |
| Phase III:  Coming off CPB | HR 100, NSR  BP 86/47  RR 15  SpO_2_ 100%  ETCO_2_ 35 | **Learner Actions:**  Learner should identify patient’s temperature.  Learner should identify patient’s rhythm and decide if pacing is necessary.  Learner should identify need to discuss heart function with staff.  Learner should identify need for inotropy/vasoconstrictor.  Learner should identify need to turn vent and monitors back to original settings.  Learner should identify hemodynamics and if they are appropriate. | **Trigger:**  Surgeon states they are ready to come off CPB  **Teaching Points:**  Discuss patient’s rhythm and potential derangements or need for pacing.  Discuss need for inotropy/vasoconstrictor based off heart function, bypass time, cardiac output. |
| Phase IV:  After CPB | HR 80 NSR  BP 100/60  RR 16  Sp0_2_ 100%  ETC0­_2_ 35 | **Learner Actions**  Learner should be able to appropriately administer protamine after informed by surgeon and anticipate possible reactions.  Learner should inform perfusionist of 50% administration of protamine.  Learner should recheck ACT after protamine administration.  Learner should check coagulation markers, thromboelastograph and platelets after ACT has normalized.  Learner should be able to check cardiac output. | **Trigger:** Hemodynamically stable and surgeon states ready for protamine  **Teaching Points:**  Discuss protamine and potential adverse reactions.  Discuss why they should announce 50% protamine administration.  Discuss possible causes of coagulation derangement including long bypass time, temperature. |

**Ideal Scenario Flow**

In this scenario, the learner is presented with an adult patient who requires cardiopulmonary bypass for aortic valve replacement. The learner will demonstrate knowledge of the basic steps to going on and coming off cardiopulmonary bypass. The learner will successfully perform the steps to go onto cardiopulmonary bypass excluding hemodynamic control. The learner should identify the need to manage glucose control and monitor urine output on cardiopulmonary bypass. The learner should have a basic plan for coming off cardiopulmonary bypass and supporting hemodynamics. The learner should appropriately identify the steps to come off cardiopulmonary bypass. Trainee may discuss the administration of heparin and protamine and possible complications associated with this. The learner may ask for help immediately and additional personnel should be made available.

**Anticipated Management Mistakes**

Learner performance will likely be contingent on level of training. It is expected that the learner not know all of the steps involved with going on and coming off cardiopulmonary bypass but instead to introduce them to the topic prior to their operating room experience. In this circumstance, desired learner actions and behavior are emphasized and discussed during verbal debriefing to further reinforce those concepts. At times, the learner may take inappropriate steps or pursue an undesirable course of action and we then either follow a best-case scenario as per the technical guide or extemporaneously modify the simulation as needed.

It would not be unexpected that junior residents struggle with this simulation; indeed this simulation can be repeated later in training to assess knowledge and skills pertaining to both the basic and more advanced components of perioperative management of Cardiopulmonary bypass. We expect that residents of greater experience level will be proficient with this simulation after they have had experience in the actual operating room setting.
