# Supplementary material for: Preparing for Cardiopulmonary Bypass: A Simulation Scenario for Anesthesia Providers
Source: MedEdPORTAL. 2017 May 8;13:10578. doi: 10.15766/mep_2374-8265.10578 (PMC6338152; doi:10.15766/mep_2374-8265.10578)
Supplement: Supplementary file 1 — A. Simulation Case.docx B. Supplemental Data.docx C. Critical Actions Checklist.docx D. Debriefing Summary.docx E. Evaluation Form.docx [file mep-13-10578-s001.zip › B. Supplemental Data.docx]

***Appendix B- Supplemental Data _ Cardiopulmonary Bypass***

Laboratory studies available upon request at outset of simulation (only give values requested):

Na: 140mEq/L

K: 4.3 mEq/L

Cl: 100 mEq/L

HC03: 28 mEq/L

BUN: 22 mg/dL

Cr: 1.2 mg/dL

Glucose: 94 mg/dL

Hemoglobin: 12.9 mg/dL

Hematocrit: 38%

Platelets: 330, 000 /mm3

ABG 7.35/35/250/28

Baseline ACT 107

Preoperative echocardiogram

| \| TRANSTHORACIC ECHOCARDIOGRAM   Enlarged left ventricle.  Mild left ventricular hypertrophy.  Normal left ventricular systolic function 55-60%.  Severe aortic valve stenosis.  Aortic valve mean gradient = 62 mmHg.  Aortic Valve Maximal Velocity = 4.7m/sec.  Aortic valve peak instantaneous gradient = 80 mmHg.  Previous aortic valve mean gradient = 47 mmHg  The estimated aortic valve area = 0.7 cm2 (continuity equation).  Mild mitral regurgitation by Doppler.  Normal cardiac catheterization \| \| --- \| \|  \| | |
| --- | --- | --- | --- |
|  |  |
|  |  |
|  |  |
|  |  |
|  |  |
|  |  |
|  |  |
|  |  |
|  |  |
|  |  |
