# Supplementary material for: Preparing for Cardiopulmonary Bypass: A Simulation Scenario for Anesthesia Providers
Source: MedEdPORTAL. 2017 May 8;13:10578. doi: 10.15766/mep_2374-8265.10578 (PMC6338152; doi:10.15766/mep_2374-8265.10578)
Supplement: Supplementary file 1 — A. Simulation Case.docx B. Supplemental Data.docx C. Critical Actions Checklist.docx D. Debriefing Summary.docx E. Evaluation Form.docx [file mep-13-10578-s001.zip › C. Critical Actions Checklist.docx]

Appendix C- Critical Actions Checklist _ Cardiopulmonary Bypass

One route for optimal management of the simulated patient takes the following steps (check as completed):

- - - Prior to Bypass
      - Baseline Activated Clotting Time (ACT)
      - Heparinization – know to withdraw blood and give heparin centrally
      - Redraw ACT at appropriate interval after heparin
      - Confirm ACT appropriate for CPB
      - Systolic Blood Pressure (SBP) <100 prior to aortic cannulation (optional)
      - Empty urometer for bypass
      - Watch circuit for clot
      - Machine Alarms to bypass mode
      - Turn Volatile gas off/ventilator off. Ensure perfusion has appropriate volatile on board
      - Turn vasoactive infusions off when Mean Arterial Pressure (MAP) stabilizes on bypass
      - Other IV anesthetics to administer?
      - Optional – verify cannulation with (Transesophageal Echocardiogram) TEE
      - Verify appropriate blood product availability
    - On bypass
      - Watch MAP, urine output, ACT
      - Optional – observe bispectral index and cerebral oximetry
      - Treat hyperglycemia
      - Do I need further coagulation testing or thromboelastogram prior to separation from bypass
      - Reassess availability/necessity for blood product
      - Patient’s heart rhythm? Pacing required? Do you have a pacing box? Did you check the batteries?
    - Coming off Bypass
      - What is patient’s temperature? Observe the difference between peripheral and core temperature
      - Check arterial blood gases, electrolytes, acid base status, hemoglobin as needed
      - Patient’s heart rhythm? Pacing required?
      - Are you ventilating?
      - Is there air in the heart on echo? Do we need a Valsalva/Trendelenburg to de-air the heart
      - Is the monitor switched off Cardiac Bypass Mode?
      - Zero your invasive pressure monitors
      - Do you have anesthetic on?
      - Inotropy? SVR? How does the heart function look on TEE? Restart vasoactive infusions as necessary
    - After Bypass
      - Separated from cardiopulmonary bypass and hemodynamics are stable, protamine?
      - Loudly announce to surgeon initiation of protamine
      - Loudly announce 50% of protamine is in. Ask surgeon permission to proceed

**Rate the trainee on the learning objectives for this case using the following statements:**

(1 is strongly disagree, 5 is strongly agree)

The Learner is able to broadly discuss the goals of cardiac bypass management

Strongly Disagree Disagree Neither Agree or Disagree Agree Strongly Agree

1 2 3 4 5

The learner is able to discuss anticoagulation strategy for the above case

Strongly Disagree Disagree Neither Agree or Disagree Agree Strongly Agree

1 2 3 4 5

The learner is able to navigate going on and off cardiopulmonary bypass

Strongly Disagree Disagree Neither Agree or Disagree Agree Strongly Agree

1 2 3 4 5
