# Supplementary material for: Preparing for Cardiopulmonary Bypass: A Simulation Scenario for Anesthesia Providers
Source: MedEdPORTAL. 2017 May 8;13:10578. doi: 10.15766/mep_2374-8265.10578 (PMC6338152; doi:10.15766/mep_2374-8265.10578)
Supplement: Supplementary file 1 — A. Simulation Case.docx B. Supplemental Data.docx C. Critical Actions Checklist.docx D. Debriefing Summary.docx E. Evaluation Form.docx [file mep-13-10578-s001.zip › D. Debriefing Summary.docx]

**Appendix D- Debriefing Summary _ Cardiopulmonary Bypass**

**General Questions:**

1. **What do you think went well?**
2. **What did you have difficulty with?**
3. **Is there anything you think you should have done differently?**

**Specific Questions:**

**4. What is the anesthesiologist’s goal for heparinization prior to CPB?**

Do not allow the surgeons to go on bypass without heparinization. If the patient is not heparinized when the clamp is opened on the bypass pump, the pump and oxygenator will clot and the patient will most likely die. If the surgeons are placing a cannula in the aorta or other large artery ask if they want the heparin given. When they ask for heparin, respond with a verbal statement - the heparin has been given. Aspirate blood from the line before and after the heparin dose to check to make sure the line is intravascular. The dose of heparin is 400 U/kg which is about 28 cc of 1000 u/cc heparin in a 70 kg man. Check the ACT three minutes after dosing. Do not use the same IV to draw the blood that you infused the heparin in. (i.e. draw an arterial blood sample). You want to check the ACT quickly because it needs to be above 450 seconds to go on bypass and that is 7.5 minutes of waiting if you forget and have not drawn the blood sample. The sample is usually drawn 3 minutes after heparin administration. If the ACT is not greater than 450 seconds after the dose, give more, until the ACT is above 450 seconds.

Add heparin to your ACLS protocol for cardiac surgery patients. If the patient suffers cardiac arrest, give the heparin so that patient can be put on bypass for resuscitation.

**5. What side-effect is common with heparinization? What’s the pathophysiology?**

Hypotension is a common side-effect of heparinization so it is sometimes helpful to administer heparin so that you have an appropriate blood pressure prior to cannulation. The suspected cause of this hypotension is from heparin binding to calcium. It is not a histamine related phenomenon.

**6. You administer heparin and give a re-dose, the ACT does not reach goal. What should you do?**

You should have a strong suspicion for antithrombin III deficiency. The management is to either give fresh frozen plasma or antithrombin III concentrate to increase the amount of AT III.

**7. Tell me about protamine and its potential complications. How do you decide on a protamine dose?**

Protamine is a peptide found in the sperm of salmon. There are allergic, anaphylactic, and histamine responses. Typically protamine dose of 10 mg will equalize Heparin 1000 units. This will vary by institution. You need to give the dose and then check the response by measuring the ACT. There is protamine in NPH insulin so there is the possibility that this will predispose individuals to have a reaction to the drug.

**Protamine Administration:** Give 10 mg = 1 cc and check for allergic response manifested as hypotension, bronchospasm, rash, or pulmonary hypertension. Our facility typically gives protamine peripherally but this is not required. Stop administration for problems. You can get severe hypotension from protamine, be ready with phenylephrine. Steroids, H1& H2 blockers, vasoconstrictors, inotropes, and returning to bypass can help. Allowing the heparin to spontaneously be metabolized is another option for severe reactions.

Then give the rest of the dose slowly. Once half of the protamine is in (specific timing at our institution), tell the perfusionist so that they can stop the pump suckers and avoid clotting the pump. This is also a great time to ask the surgeon if he is alright with finishing protamine administration. If you clot the pump and need to return to bypass you may not be able to.

Once all the protamine is in, inform the surgeons, and then check an ACT. You should return to baseline (120 - 130). If you have not, give more protamine. If you give pump blood after this point you may need to give more protamine. After a normal ACT, check you coagulation system with normal markers, TEG and platelet level.

**8. Pneumonic for Going on Bypass:**

**HAD2SUE**

**H**eparin: Always give prior to bypass.

**A**CT: Always check before going on bypass (450 seconds)

**D**rugs: Do you need anything (Non-depolarizing neuromuscular blocker).

**D**rips: Turn off the inotropes etc.

**S**wan: Pull the PA catheter back 5 cm to avoid pulmonary arterial occlusion/rupture.

**U**rine: Account for bypass urine

**E**mboli: Check the Arterial cannula for bubbles.

**9. Pneumonic for Getting Off Bypass:**

**WRMVP:** Wide receiver most valuable player.

**W**arm: What is the bladder and blood temp?

**R**hythm: Are they in NSR or do you need to pace? Is the rate adequate?

**M**onitors On: Turn them back on if you turned them off for bypass. Turn back on the alarms.

**V**entilation: Turn on the ventilator. Easy to forget and you look very silly.

**P**erfusion: What is the pump flow?

**References**

Wallace, A. (2011). Cardiac Anesthesiology Made Ridiculously Simple. Retrieved March 1, 2016, from http://www.cardiacengineering.com/cardiaca.htm
